# Reassessing the prognostic value of station 108 in the era of neoadjuvant therapy

**DOI:** 10.1097/JS9.0000000000003071

**Published:** 2025-07-14

**Authors:** Wei Wei, Yihang Yuan

**Affiliations:** a Department of Esophageal Surgery, Department of Thoracic Surgery, Nanjing Drum Tower Hospital, the Affiliated Hospital of Nanjing University Medical School, Nanjing, China; b Department of General Surgery, Nanjing Drum Tower Hospital, the Affiliated Hospital of Nanjing University Medical School, Nanjing, China

*Dear Editor,*

We read with great interest the recent study by Jiang and colleagues published in the *International Journal of Surgery*, which provides a valuable contribution to the understanding of esophageal squamous cell carcinoma (ESCC) prognosis by identifying station 108 metastasis as an independent predictor of poor overall survival (OS) and disease-free survival (DFS)^[^1^]^. However, several important aspects warrant further discussion, especially considering recent advances in multimodal treatment strategies for ESCC. This correspondence is compliant with the TITAN Guidelines 2025—governing declaration and use of AI^[^2^]^.

A major point deserving clarification is the study’s focus on patients undergoing surgery alone, without neoadjuvant therapy. While this approach eliminates potential confounding effects of tumor downstaging, it may limit the generalizability of findings in contemporary clinical practice, where neoadjuvant chemoradiotherapy (NCRT) or chemoimmunotherapy is increasingly adopted as standard of care for locally advanced ESCC^[^3^]^. Neoadjuvant therapy fundamentally changes the biological and pathological characteristics of lymph nodes (LNs), often leading to a reduction in metastasis frequency and altered distribution patterns^[^4^]^. For example, in a recent study of middle and lower thoracic ESCC patients treated with NCRT followed by minimally invasive esophagectomy, the overall LN metastasis rate was as low as 4.1%. Notably, in that study, while dissection of ≥15 LNs improved survival outcomes, LN metastases were not significantly associated with OS or DFS^[^5^]^. These findings appear to contrast with those of Jiang et al.

In addition, the limited sensitivity and specificity of conventional imaging modalities such as contrast-enhanced CT in detecting LN metastases poses a barrier to preoperative risk stratification. If station 108 is indeed prognostically critical, then its radiologically occult nature poses a significant challenge for personalized treatment planning. Future studies should explore the utility of advanced imaging techniques, such as dual-energy CT or MRI^[^6^]^, in the accurate identification of station 108 involvement.

In conclusion, future studies should investigate whether the prognostic relevance of station 108 metastasis is maintained following neoadjuvant therapy. Moreover, integrating advanced imaging modalities into preoperative assessments may enable more refined risk stratification and facilitate truly individualized treatment pathways for patients with ESCC.

## Data Availability

All data are included in the article.
